# Highlights in Virology: Viral miRNAs and Flaviviruses

**DOI:** 10.3389/fcimb.2019.00054

**Published:** 2019-02-26

**Authors:** Lawrence Banks

**Affiliations:** International Centre for Genetic Engineering and Biotechnology, Trieste, Italy

**Keywords:** virology, flaviviruses, miRNA, viral, cancer

Despite being discovered over 50 years ago, and its close association with the development of several important human cancers being well-documented, Epstein Barr Virus (EBV) continues to be a source of important information on how viruses can contribute toward the development of human cancer. EBV is known to be an important carcinogen, but because of its large genome (around 172 kb), much still remains to be discovered about its normal viral life cycle and how it drives malignancy at different anatomical sites.

In a recent study in PLoS Pathogens (Lyu et al., 2018) Lyu et al. report, not on a viral protein, but on a viral miRNA, and how that can contribute toward the development of malignancy. Indeed, EBV has been a rich source of virally-derived miRNAs, the activities of which have been linked to tumor development. In this particular study, the authors have found that one specific EBV miRNA, EBV-miR-BART1-5P, plays an important role in promoting glycolysis and angiogenesis in nasopharyngeal carcinoma cells, which is mediated by directly targeting AMP-activated protein kinase 1 and thereby deregulating the AMPK/mTOR pathway.

Studies such as this are exciting because they begin to define how these viral miRNAs can both contribute toward malignant development and provide favorable environments for viral replication. It will now be very interesting to determine whether blocking the function of these miRNAs has any potential therapeutic applications.

In contrast to EBV, two viruses that have only more recently gained notoriety are Dengue and Zika, which belong to the family of Flaviviruses. These viruses are mosquito-borne and represent major and growing health problems in many parts of the world. Whilst a great deal of attention has been placed on understanding the immunology underlying clearance of infection with these viruses, much less is known about the underlying mechanisms by which the viral gene products interact with components of the host cell.

In a proteomic tour de force published in the recent issue of Cell (Shah et al., 2018), Shah et al. have defined the virus-host protein interactome for Dengue and Zika in their two hosts, the mosquito and the human. A gem from this study was the indication that the Zika NS4A protein interacts with a cellular protein linked to the development of microcephaly. Notwithstanding this exciting observation, this study represents an amazing resource for any groups wishing to further characterize the molecular bases for the different biological outcomes associated with infection by these two Flaviviruses.

Since Shah et al. provide data on both the mosquito and human viral interactomes, this opens the way for studies to specifically characterize the functions of different viral proteins in the two different hosts and may offer leads for the development of novel therapies or preventative measures.

## Author Contributions

The author confirms being the sole contributor of this work and has approved it for publication.

### Conflict of Interest Statement

The author declares that the research was conducted in the absence of any commercial or financial relationships that could be construed as a potential conflict of interest.
